# Ring Pressure Sign: When Long Lasting Dermoscopic Observation Leads the Decision

**DOI:** 10.5826/dpc.1104a125

**Published:** 2021-10-01

**Authors:** Vincenzo Piccolo, Teresa Russo, Giuseppe Argenziano

**Affiliations:** Dermatology Unit, University of Campania “Luigi Vanvitelli”, Naples, Italy

**Keywords:** ring pressure sign, dermoscopy, dermatoscopy, melanoma, mole check

Dermoscopy is an indispensable tool for early detection of skin cancer. Beyond that, its widespread use can be attributed to the fact that dermoscopy is not time consuming. Indeed, experienced dermoscopists take less than 1 second to analyze each of their patients’ lesions through a handheld dermoscope. This is possible due to prompt (‘gestalt’) cerebral recognition of the overall appearance of the lesion, as benign or suspicious, which does not require a detailed pattern analysis. The so-called “blink” approach allows for a rapid check of most lesions, although some cases require more time to be diagnosed and a “think” approach is necessary to take a decision [1,2]. Indeed, there are cases in which the dermoscopist occasionally stops and takes time to examine a single lesion, sometimes requiring up to one minute of examination. In cases that require longer observation times, when using a contact dermoscope, a ring might appear around the observed lesion. This is due to the dermoscope pressure exerted on the patient’s skin (Figures 1 and 2).

While clear-cut benign or malignant lesions do not require long time to be detected, doubtful lesions are responsible for the appearance of the so-called “ring pressure sign”, which in most cases leads to excision. This sign is probably due to our inability to promptly categorize the lesion as benign or malignant; while not an absolute sign, it may be considered a good indicator for the dermoscopist’s suspicion.

## Figures and Tables

**Figure 1:**
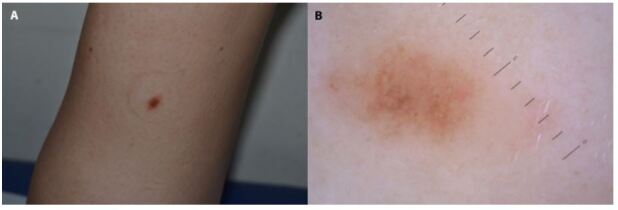
(A) A ring caused by pressure due to the long observation through contact dermoscopy is seen in this young female with this not clear cut malignant lesion. Histology showed melanoma in situ. (B) Dermoscopy showed in the context of light brown background a delicate irregular network.

**Figure 2:**
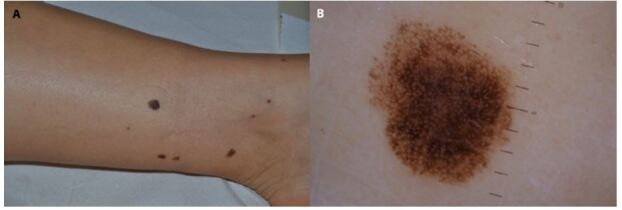
(A) “Ring pressure sign” determined by prolonged pressure of contact dermoscope in a 23-year-old woman, whose lesion was excised and melanoma in situ was diagnosed at histology. (B) Pigmented network with sharp demarcation at periphery, that showed irregularity concerning thickness at dermoscopy.
